# Occlusion type and posterior communicating artery patency may predict favorable outcome after endovascular thrombectomy in selective basilar top occlusion

**DOI:** 10.3389/fneur.2022.1047971

**Published:** 2022-11-17

**Authors:** Jung Soo Park, Yong-Jin Im, Byoung-Soo Shin, Hyun Goo Kang

**Affiliations:** ^1^Research Institute of Clinical Medicine of Jeonbuk National University Biomedical Research Institute of Jeonbuk National University Hospital, Jeonju, South Korea; ^2^Department of Neurosurgery, Jeonbuk National University Medical School and Hospital, Jeonju, South Korea; ^3^Center for Clinical Pharmacology and Biomedical Research Institute, Jeonbuk National University Hospital, Jeonju, South Korea; ^4^Department of Neurology, Jeonbuk National University Medical School and Hospital, Jeonju, South Korea

**Keywords:** basilar artery, ischemic stroke, outcome, posterior communicating artery, thrombectomy

## Abstract

**Introduction:**

The top of the basilar artery is a five-branched junction, consisting of two superior cerebellar arteries (SCAs), two posterior cerebellar arteries (PCAs), and the basilar artery itself. This study aimed to investigate prognostic factors in patients with selective acute basilar top occlusion managed with mechanical thrombectomy, focusing on occlusion type and posterior communicating artery (PCoA) patency.

**Methods:**

Eligible patients who underwent endovascular treatment (EVT) for acute basilar top occlusion were reviewed. Patterns of basilar top occlusion were categorized as types I–III according to whether the SCA and PCA were visible on angiography. The PCoA was categorized as hypoplastic or non-visible (type I), normal patency but non-visible PCA through PCoA flow (type II), and fetal type (type III).

**Results:**

Good outcomes were observed in 50% (21/42) and mortality in 11.9% (5/42) of the cases at 90 days. Patients with good outcomes showed lower baseline National Institutes of Health Stroke Scale (NIHSS) score (*P* = 0.001) and a higher proportion of type III basilar top occlusion (*P* = 0.004) and type III PCoA (*P* = 0.001). Multivariable logistic analysis showed that baseline NIHSS score [odds ratio (OR), 0.84; 95% confidence interval (CI), 0.73–0.97; *P* = 0.017) and type III PCoA (OR, 21.54, 95% CI, 1.33–347.97; *P* = 0.031) were independent predictors of good functional outcomes.

**Conclusion:**

A low initial NIHSS score and good PCoA patency were independent predictors of favorable clinical outcomes after EVT in patients with acute basilar top occlusion. Furthermore, the favorable outcome group showed a high proportion of type III basilar top occlusions.

## Introduction

The distal basilar artery (BA) is a five-branched junction comprising two superior cerebellar arteries (SCAs), two posterior cerebral arteries (PCAs), and one BA. Acute occlusion of this area is described as “top of the basilar syndrome” (TOBS) and may include infarction of the occipital lobe, thalamus, cerebellum, and rostral midbrain (1, 2). In the past, TOBS was diagnosed on the basis of clinical symptoms and lesion distribution. However, recent radiologic confirmation of TOBS has determined a diagnosis based on the correlation between the actual occlusion site and definition of the disease entity through catheter angiography during endovascular treatment (EVT) in selective patients.

The symptoms of TOBS are usually fatal and involve severe mental deterioration, while some patients present with minimal or atypical symptoms (3–5). The various clinical features may be due to the diverse pattern of basilar top occlusion, and collateral flow from the anterior circulation through the posterior communicating artery (PCoA) (6). Thus, we aimed to investigate prognostic factors in patients with selective basilar top occlusion treated with mechanical thrombectomy, focusing on categorized occlusion type based on lesion distribution, and PCoA based on the flow pattern.

## Materials and methods

### Study design and patients

We identified patients with acute ischemic stroke treated with EVT between January 2012 and November 2019. Our inclusion criteria targeted patients with basilar top occlusion confirmed by catheter angiography during EVT. As specifying the extent of the thrombus burden is difficult when the bilateral SCAs are not visible, basilar top occlusion was defined as a case in which at least one SCA was visible. During this period, 930 patients with acute ischemic stroke underwent EVT. Among them, 888 patients were excluded according to the following subcategories: 801 patients without posterior circulation occlusion, 59 with bilateral SCAs not visible on catheter angiography, six with distal PCA occlusion (distal to the P2 portion of the PCA), 13 with vertebral artery orifice occlusion, and nine with concomitant anterior circulation occlusion. The final sample comprised 42 patients (shown in Supplementary Figure 1).

### Endovascular treatment

Patients received either bridging therapy (EVT combined with intravenous thrombolysis) or EVT alone. All eligible patients were given 0.9 mg/kg of intravenous recombinant tissue plasminogen activator within 4.5 h of symptom onset. The manual aspiration technique with either a Penumbra aspiration catheter (Penumbra, Alameda, CA, USA) or SOFIA catheter (MicroVention, Inc., Tustin, CA, USA) was used as first-line EVT. If recanalization was not achieved with this technique, stent retrieval was performed using the Solitaire stent system (Covidien, Irvine, CA, USA). All patients underwent bilateral ICA angiography to assess PCoA patency after EVT. Recanalization was assessed using post-procedure angiography, and successful recanalization was defined as a modified thrombolysis treatment in cerebral infarction (TICI) scale score of 2b-3.

### Clinical and imaging analysis

**Figure 1:**
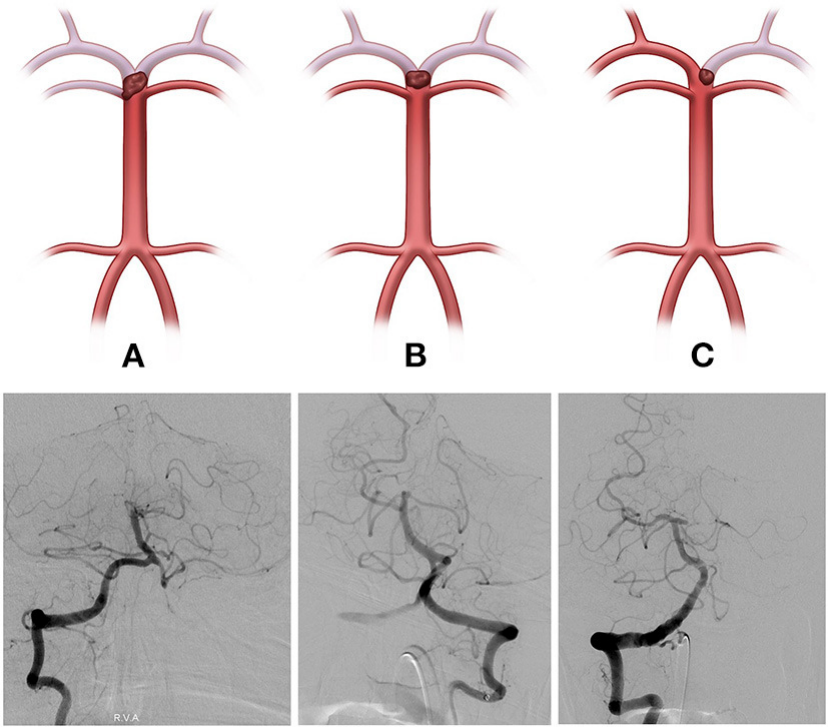
The basilar top occlusion type was classified into three groups (Illustrations and angiographies). (**A**, Type I) Bilateral posterior cerebral arteries (PCA), with one side of the superior cerebellar artery (SCA) not visible on catheter angiography, (**B**, Type II) Bilateral PCA not visible with both SCA intact, and (**C**, Type III) PCA occlusion on one side.

**Figure 2:**
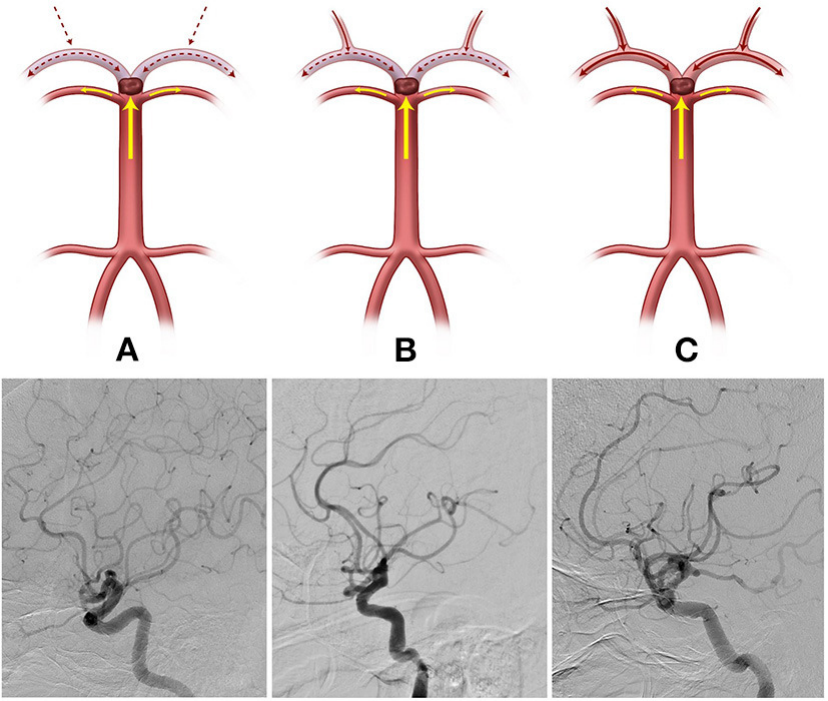
The posterior communicating artery (PCoA) types were categorized into three groups (Illustrations and angiographies). (**A**, Type I) Hypoplastic or non-visible PCoA, (**B**, Type II) Normal patency but PCA not visible through PCoA flow on the internal carotid artery angiogram, and (**C**, Type III) Good patency with visible PCA or fetal-type PCA.

The initial neurological status of each patient was evaluated using the National Institutes of Health Stroke Scale (NIHSS) score at admission. The modified Rankin scale (mRS) score was checked at admission and at 3 months for all patients, and a favorable functional outcome was defined as 3-month mRS score ≤ 2. The basilar top occlusion type was classified into the following three groups: bilateral PCA with one side of the SCA not visible on catheter angiography (type I), bilateral PCA not visible with an intact SCA (type II), and one side of PCA occluded (type III; shown in Figure 1). PCoA was categorized as hypoplastic or non-visible PCoA (type I), normal patency but no visible PCA through PCoA flow on internal carotid artery angiogram (type II), and good patency with visible PCA or fetal-type PCA (type III; shown in Figure 2).

### Statistical analysis

We compared the demographics, clinical, and procedural data of the patients receiving EVT. We used Pearson's chi-square or Fisher's exact tests to analyze the categorical variables, and Student's *t*-test for continuous variables. Continuous variables are summarized as means with standard deviations or medians with interquartile ranges, while categorical data have been expressed as counts and percentages. We then performed a multivariate analysis to identify the basilar top occlusion type, PCoA flow pattern, and other variables as independent factors for good functional outcomes. To avoid variable selection caused by spurious correlations, we included only variables potentially associated with mRS score ≤ 2 on univariate analysis (*P* < 0.1) as the potential factor associated with favorable outcomes in the multivariate logistic regression model. Statistical significance was set at *P* < 0.05, and SPSS (version 21.0; IBM Corporation, Armonk, NY, USA) was used for all statistical analyses.

## Results

The mean age of the 42 eligible patients was 68.1 ± 14.4 years, and 30 (71.4%) of them were male. The median (interquartile range) baseline NIHSS score was 12.5 (range, 8–20.25), and the median (interquartile range) discharge NIHSS score was 10 (range, 2–18.25). In this study, the proportions of basilar top occlusion types were 26.2% for type I (11 patients), 42.9% for type II (18 patients), and 31.0% for type III (13 patients). Type I (hypoplastic or non-visible PCoA) was observed in 10 patients (23.8%), type II (normal PCoA but non-visible PCA) in 21 patients (50%), and type III (good patency or fetal-type PCA) in 11 patients (26.2%). Among all patients, 24 (57.1%) underwent bridging therapy [EVT combined with intravenous (IV) thrombolysis], whereas the remaining 18 (42.9%) received only EVT. Following EVT, 78.6% of patients (33/42) showed successful recanalization (TICI 2b-3). Among them, 42.8% (18/42) had atrial fibrillation, and the prevalence of symptomatic intracranial hemorrhage within 24 h was 2.4%. Overall, favorable outcomes were achieved in 21 (50%) of the 42 patients, and mortality was encountered in 11.9% (5/42) of the patients at 90 days.

The baseline patient characteristics are shown in Table 1. Patients with favorable outcomes had a lower baseline NIHSS score than those with poor outcomes (10 vs. 18, *P* = 0.001); a greater proportion had type III basilar top occlusion [52.4% (11/21) vs. 9.5% (2/21), *P* = 0.004] and type III PCoA pattern [47.6% (10/21) vs. 4.8% (1/212), *P* = 0.001]. No significant differences were observed in the frequency of recombinant tissue plasminogen activator use or stroke risk factors. Regarding procedural and clinical data related to EVT, the favorable and poor outcome groups were not significantly different (Table 2). Multivariable logistic analysis showed that baseline NIHSS score [odds ratio (OR), 0.84; 95% confidence interval (CI), 0.73–0.97; *P* = 0.017] and type III PCoA (OR, 21.54; 95% CI, 1.33–347.97; *P* = 0.031) were independent predictors of good functional outcomes (Table 3). Furthermore, upon successful recanalization (TICI 2b-3) after EVT, the onset-to-door time was longer (106.67 ± 54.31 vs. 199.39 ± 149.23, *P* = 0.006), while the proportion of patients with atrial fibrillation was significantly higher (11.1 vs. 51.5%, *P* = 0.030, Table 4).

**Table 1:** Comparison of baseline characteristics between patients with favorable (mRS ≤ 2) and poor (mRS >2) outcomes.

	**Favorable outcome**	**Poor outcome**	***P*** **value**
	**(*n =* 21)**	**(*n =* 21)**	
Age, years	67.4 (13.9)	68.9 (15.1)	0.752
Male	17 (81.0)	13 (61.9)	0.172
Baseline NIHSS score	10 (6–12.5)	18 (12.5–23.5)	0.001
Discharge NIHSS score	2 (1–6)	18 (12–31.5)	< 0.001
**Occlusion type**			
Type I	2 (9.5)	9 (42.9)	
Type II	8 (38.1)	10 (47.6)	0.004
Type III	11 (52.4)	2 (9.5)	
**PCoA type**			
Type I (hypoplastic or non-visible)	1 (4.8)	9 (42.9)	
Type II (normal)	10 (47.6)	11 (52.4)	0.001
Type III (fetal-type)	10 (47.6)	1 (4.8)	
IV t-PA use	14 (66.7)	10 (47.6)	0.229
Risk factors			
Diabetes mellitus	4 (19.0)	4 (19.0)	1
Hypertension	12 (57.1)	16 (76.2)	0.190
Hyperlipidemia	2 (9.5)	3 (14.3)	1
Smoking	7 (33.3)	6 (28.6)	1
Previous stroke history	1 (4.8)	4 (19.0)	0.343
Atrial fibrillation	11 (52.4)	7 (33.3)	0.212

Data are expressed as numbers (% column), means (SD), or medians (25th−75th percentile range). Non-parametric tests were performed for continuous variables that did not show normal distribution, and are presented as medians (25th−75th percentile range). PCoA, posterior communicating artery; NIHSS, NIH Stroke Scale; mRS, modified Rankin scale; t-PA, tissue plasminogen activator.

**Table 2:** Comparison of procedural and clinical data between patients with favorable (mRS ≤ 2) and poor (mRS >2) outcomes.

	**Favorable outcome**	**Poor outcome**	***P*** **-value**
	**(*n =* 21)**	**(*n =* 21)**	
Mean procedure time (min)	22 (13–29)	30 (19–43.5)	0.151
Onset-to-door time (min)	150 (90–180)	150 (60–360)	0.171
Onset to recanalization (min)	308 (200–433.5)	375 (289–488)	0.154
Successful recanalization (TICI 2b-3)	17 (81.0)	16 (76.2)	1
Symptomatic ICH	0 (0)	1 (4.8)	1
Onset to recanalization < 6 h	12 (57.1)	8 (38.1)	0.217
Onset to recanalization < 12 h	20 (95.2)	19 (90.5)	1

Data are expressed as numbers (% column), means (SD), or medians (25th−75th percentile range). Non-parametric tests were performed for continuous variables that did not show normal distribution, and are presented as medians (25th−75th percentile range). ICH, intracerebral hemorrhage; TICI, thrombolysis in cerebral infarction; mRS, modified Rankin scale.

**Table 3:** Factors associated with good functional outcome (mRS 0–2 at 3 months).

	**Crude OR (95% CI)**	***P*** **-value**	**Adjusted OR (95% CI)**	***P*** **value**
Baseline NIHSS score	0.84 (0.75–0.95)	0.004	0.84 (0.73–0.97)	0.017
Basilar top occlusion (type III)	10.45 (1.93–56.64)	0.007	2.45 (0.27–22.60)	0.430
PCoA (type III)	18.18 (2.05–161.38)	0.009	21.54 (1.33–347.97)	0.031

Data are expressed as ORs and 95% CIs. Variables with *P* < 0.1 by univariate analysis were entered into the multivariate analysis model. PCoA, posterior communicating artery; OR, odds ratio; CI, confidence interval; NIHSS, NIH stroke scale; mRS, modified Rankin scale.

**Table 4:** Comparison of baseline characteristics in those with and without successful recanalization (TICI 2b-3).

	**TICI < 2b (*n =* 9)**	**TICI ≥2b (*n =* 33)**	***P*** **-value**
Age, years	66.00 ± 18.22	68.73 ± 13.40	0.620
Male	7 (77.8)	23 (69.7)	0.634
Baseline NIHSS score	15 (10–24)	12 (7.5–18.5)	0.296
Discharge NIHSS score	12 (2–20)	8 (2.5–16)	0.775
**Occlusion type**			
Type I	1 (11.1)	10 (30.3)	
Type II	3 (33.3)	15 (45.5)	0.176
Type III	5 (55.6)	8 (24.2)	
**PCoA type**			
Type I (hypoplastic or non-visible)	2 (22.2)	8 (24.2)	
Type II (normal)	4 (44.4)	17 (51.5)	0.858
Type III (fetal-type)	3 (33.3)	8 (24.2)	
**Risk factors**			
Diabetes mellitus	1 (11.1)	7 (21.2)	0.494
Hypertension	5 (55.6)	23 (69.7)	0.425
Hyperlipidemia	2 (22.2)	3 (9.1)	0.281
Smoking	3 (33.3)	10 (30.3)	0.862
Previous stroke history	1 (11.1)	4 (12.1)	0.934
Atrial fibrillation	1 (11.1)	17 (51.5)	0.030
Mean procedure time (min)	32.44 ± 15.68	26.06 ± 14.16	0.248
Onset-to-door time (min)	106.67 ± 54.31	199.39 ± 149.23	0.006
Onset to recanalization (min)	310.78 ± 82.41	392.70 ± 203.00	0.247
Symptomatic ICH	0 (0)	1 (3.0)	0.597

Data are expressed as numbers (% column), means (SD), or medians (25th−75th percentile range). Non-parametric tests were performed for continuous variables that did not show normal distribution, and are presented as medians (25th−75th percentile range). PCoA, posterior communicating artery; TICI, thrombolysis in cerebral infarction; NIHSS, NIH stroke scale; ICH, intracerebral hemorrhage.

## Discussion

TOBS was first reported by Caplan (1), referring to symptoms caused by circulatory disturbances occurring at the top of the basilar artery, consisting of the junction of five blood vessels (two SCAs, two PCAs, and one BA) (1). In clinical practice, TOBS tends to be referred to as distal BA occlusions and the various symptoms caused by them rather than the proper definition of Caplan. Unlike previous studies, the present study excluded brain stem infarction due to the perforator of the BA, such as pons or medullary infarction, among patients who were admitted owing to acute BA occlusion and underwent EVT in order to target only patients with actual TOBs, which are the five blood vessels junction. This study defined patients with occlusion at the junction of the five blood vessels as TOBS patients and analyzed their prognoses.

The BA is 25–35 mm long, and the diameter is 2.7–4.3 mm (7). It is a delicate intracranial blood vessel that tapers distally (7). TOBS can lead to various outcomes; anatomically, many arteries branch off the BA. Therefore, the extensiveness of the thrombus blocking the BA varies, and the state of collateral circulation, including PCoA, is different. The collateral flow that occurs with an occluded BA, is affected by the extent of the thrombotic occlusion. Concerning BA occlusion type I in this study, the SCA and both ends of the PCA were occluded; thus, the blood flow in most vessels branching from the basilar tip was obstructed. Conversely, in the case of BA occlusion type III, a certain portion of the blood flow in the vessels originating from the basilar top is maintained through branches from both SCAs and one PCA. Considering this possibility, we classified BA occlusions into three types and evaluated the differences in prognosis. The favorable outcome group had more type II than type I, and type III accounted for the highest proportion. However, in the poor outcome group, type III was the least common, whereas types I and II showed higher proportions (*P* = 0.004, Table 1). The association between BA occlusion type and good outcomes can be ascertained in a larger cohort in the future.

PCA supplies blood to a large territory derived from the mesencephalic, diencephalic, and telencephalic embryonic structures. PCA generally results from the terminal bifurcation of the BA, which receives blood supply from the BA in 70% of cases, from the PCoA in 20% of cases, as well as from both the BA and PCoA, the so-called mixed type, constituting 10% of cases (8). Fetal-type PCA is a cerebrovascular variant that is found in 16–40% of the population (9), defined as a case in which the P1 segment of the PCA (which is between the top of the BA and PCoA) is hypoplastic or absent, as a continuous flow with PCoA is concurrently created by the P2 segment of PCA (10). We believe that despite BA occlusion, the degree of PCA flow could differ depending on the presence of the PCA variant and difference in the collateral status, and these differences may eventually affect the patient's prognosis. Therefore, we classified the PCoA types into three groups to distinguish differences in the flow of PCA by PCoA. We evaluated whether the patient outcomes varied according to the degree of PCA flow. As expected, the frequency of PCoA type III, which refers to fetal-type PCA or a good collateral status of PCoA, was high in the group with favorable outcomes, whereas it was very low in the group with poor outcomes (*P* = 0.001, Table 1). Moreover, the results of the multivariate analysis reveal PCoA type III as an important factor for good functional outcomes in TOBS (OR, 21.54; 95% CI, 1.33–347.97, *P* = 0.031; Table 3).

The rate of artery occlusion and degree of collateral status may vary depending on the mechanism of BA occlusion. BA occlusion due to an embolic source, occurs acutely, and tends to recanalize well during EVT (11). Conversely, occlusion due to atherothrombosis can progress relatively slowly to cause collateral development (11). Although BA occlusion and PCoA types were not significantly different in this study, several studies have reported that good posterior circulation collateral score was independent predictor of good outcome in patients with acute basilar artery occlusion treated with EVT. In particular, in the scoring system that evaluates the collateral status of the posterior circulation, such as basilar artery on computed tomography angiography (BATMAN) score or posterior circulation collateral score (PC-CS), the patency of PcoA was given twice the weight of other areas. (12, 13) And also, more patients had atrial fibrillation, even though the onset-to-door time was longer in successful recanalization (TICI 2b-3) cases (Table 4). Considering this result, in the case of occlusion due to a cardioembolic source, it could recanalize relatively well compared to other occlusion etiology (Table 4). Further large-scale studies are needed to address this topic.

This study had several limitations. First, it entailed a potential selection bias as the data were collected and retrospectively analyzed. Second, the overall sample size was small because only a few patients had an acute BA occlusion. Thus, it was difficult to include several variables in the multivariate analysis. Therefore, a well-designed study focusing on this issue is necessary. Third, classifications according to stroke etiology, including underlying BA stenosis, were not analyzed. This difference in stroke etiology may have influenced the prognosis and recanalization rates of patients with TOBS. This hypothesis can be confirmed in future large-scale studies.

The baseline NIHSS score and good PCoA patency (type III PCoA) can predict good prognoses after IV thrombolysis with or without EVT in patients with acute TOBS. Moreover, although not a predictor of good prognosis, the proportion of BA distal occlusion (occlusion type III) was significantly higher in the good prognosis group than in the poor outcome group. Although these results may not provide specific insights into the decision-making process involved in treating patients with acute TOBS, they will assist clinicians in predicting prognoses after treatment and preparing appropriate treatment based on the situation.

## Data availability statement

The original contributions presented in the study are included in the article/Supplementary material, further inquiries can be directed to the corresponding author.

## Ethics statement

The studies involving human participants were reviewed and approved by the Jeonbuk National University Hospital Institutional Review Board (CUH 2022-02-036). The patients/participants provided their written informed consent to participate in this study.

## Author contributions

JSP and HGK: conceptualization and writing—review and editing. JSP and B-SS: methodology and writing—review and editing. JSP, HGK, B-SS, and Y-JI: formal analysis and investigation. HGK: funding acquisition. B-SS: supervision. All authors contributed to the article and approved the submitted version.

## Funding

This research was supported by National University Development Project at Jeonbuk National University in 2021.

## Conflict of interest

The authors declare that the research was conducted in the absence of any commercial or financial relationships that could be construed as a potential conflict of interest.

## Publisher's note

All claims expressed in this article are solely those of the authors and do not necessarily represent those of their affiliated organizations, or those of the publisher, the editors and the reviewers. Any product that may be evaluated in this article, or claim that may be made by its manufacturer, is not guaranteed or endorsed by the publisher.
