# Supplementary figures and images for: Occlusion type and posterior communicating artery patency may predict favorable outcome after endovascular thrombectomy in selective basilar top occlusion
Source: Front Neurol. 2022 Nov 17;13:1047971. doi: 10.3389/fneur.2022.1047971 (PMC9716350; doi:10.3389/fneur.2022.1047971)

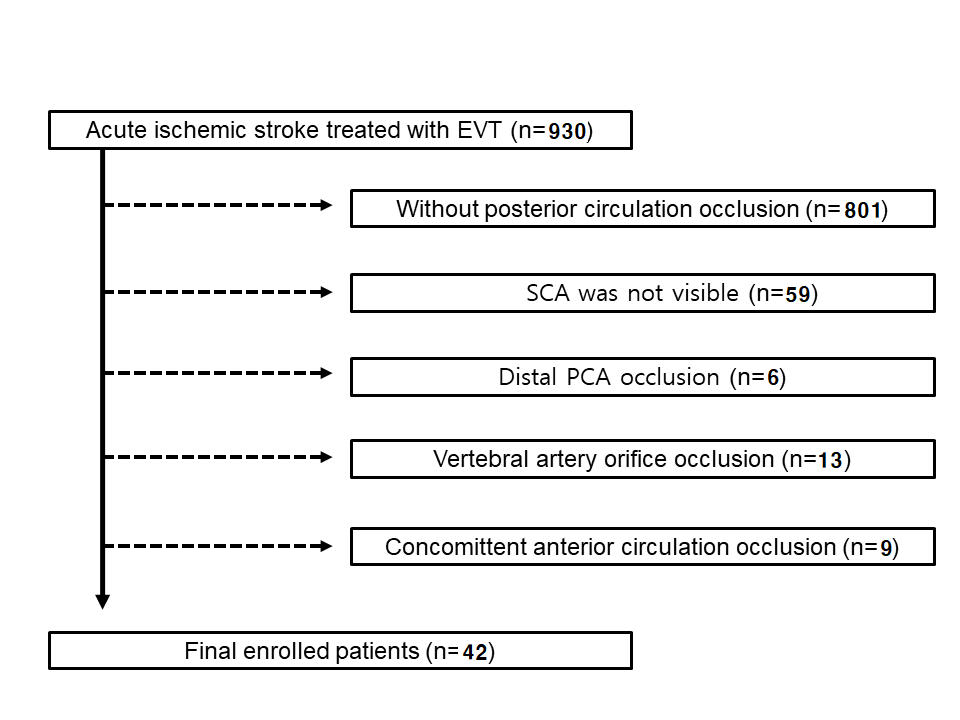

Supplement: Supplementary file 1 [file Image_1.tif]
